# Hybrid Electro-Optical Pumping of Active Plasmonic Nanostructures

**DOI:** 10.3390/nano10050856

**Published:** 2020-04-29

**Authors:** Andrey A. Vyshnevyy, Dmitry Yu. Fedyanin

**Affiliations:** Laboratory of Nanooptics and Plasmonics, Moscow Institute of Physics and Technology, Dolgoprudny 141700, Russia or

**Keywords:** active plasmonics, surface plasmon amplification, optical pumping, electrical pumping, Schottky barrier diode, plasmonic amplifier, metal-semiconductor structures

## Abstract

Surface plasmon polaritons (SPPs) offer a unique opportunity to overcome the diffraction limit of light. However, this opportunity comes at the cost of the strong absorption of the SPP field in a metal, which unavoidably limits the SPP propagation length to a few tens of micrometers in nanostructures with deep-subwavelength mode confinement. The only possibility to avoid the propagation losses is to compensate for them by optical gain in the adjacent active medium. Different approaches for surface plasmon amplification by stimulated emission of radiation have been proposed based on either optical or electrical pumping. However, each has its own disadvantages caused by the selected type of pumping scheme. Here, we study, for the first time, hybrid electro-optical pumping of active plasmonic waveguide structures, and by using comprehensive self-consistent numerical simulations, demonstrate that this hybrid approach can outperform both pure electrical pumping and pure optical pumping. The SPP modal gain is higher than under pure optical pumping, while one can precisely and locally adjust it by tuning the electric current, which allows the reduction of amplification noise and provides additional functionalities. We believe that our findings lay a solid foundation for the development of a new generation of active plasmonic devices and stimulate further research in this area.

## 1. Introduction

Surface plasmon polaritons (SPPs), which are surface electromagnetic waves at the interface between a metal and a dielectric, give a unique opportunity to design sub-diffraction limited optical components and devices [1,2]. However, the ability to harness the benefits offered by plasmonics is severely limited by losses in the metal, which is an essential part of most plasmonic devices. These losses limit the SPP propagation length to a few tens of micrometers [3,4,5]. The problem is particularly pronounced in deep-subwavelength plasmonic waveguides, where the portion of the electromagnetic field of the SPP in the metal becomes comparable with that in the surrounding dielectrics. To overcome this problem, massive efforts are being spent on improving the optical properties of plasmonic metals [6,7,8,9,10] and finding novel waveguide configurations that enable strong field confinement at moderate propagation losses [11,12,13]. However, these measures can mitigate the loss problem only to a limited extent. Therefore, active compensation of the SPP propagation losses is a necessity [14,15,16]. 

Loss compensation is based on the stimulated emission of SPP quanta into the plasmonic mode from a gain medium, which can be pumped either optically [16,17,18,19,20] or electrically [21,22,23]. The ultimate goal is to fully compensate for the SPP propagation losses, which gives the possibility to transmit optical signals over long distances (~1 cm) via deep-subwavelength waveguides [24] and design truly nanoscale light sources [25,26,27,28]. Significant progress has been made in achieving this goal using optical pumping. It was demonstrated that using a gain medium placed near the metal surface, one can fully compensate for the SPP propagation losses [18,20], and moreover, create a significant net SPP modal gain, which allows to obtain lasing of plasmonic modes [19,29,30,31]. Although optical pumping can be easily implemented in a laboratory, it is impractical due to the very poor scalability and low energy efficiency. At the same time, electrical pumping is scalable and efficient. However, surface plasmon amplification under electrical pumping is more challenging, since it is more difficult to create a high material gain in the active medium due to self-heating effects [32] and problems with contacts. Plasmonic metals (Ag, Au, Cu, and Al) do not form ohmic contacts to direct-bandgap semiconductors, which are used as electrically pumped gain media [33,34], while ohmic contacts to these semiconductors (typically based on titanium and chromium) cannot be used to guide SPPs due to extremely high losses. These issues greatly complicate the design of electrically pumped active plasmonic structures that are capable of full compensation for the SPP propagation losses [23], while partial SPP loss compensation is significantly easier to achieve using traditional waveguide and resonator geometries [35].

In this work, we study, for the first time, hybrid electro-optical pumping of active plasmonic waveguide structures, which combines the strengths of the pure electrical and pure optical techniques, and demonstrate that such a hybrid approach provides improved performance and additional functionalities.

## 2. Results and Discussion

**Figure 1:**
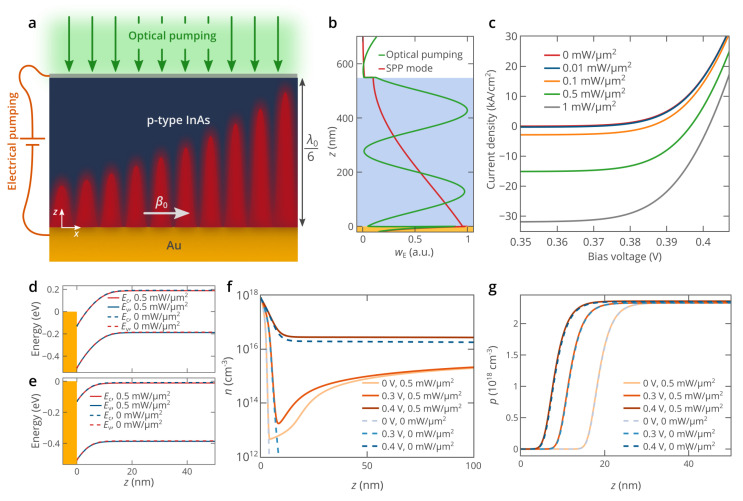
(**a**) Schematic illustration of surface plasmon polariton amplification in an electro-optically pumped active planar plasmonic waveguide, *β*_0_ is the SPP wavevector and *λ*_0_ is the free-space wavelength at which the SPP is excited. (**b**) Spatial distributions of the electric energy density of the SPP mode and the optical pump wave. The SPP is excited at a free space wavelength of 3.26 μm (*ε*_Au_ = −561 + 30*i* [24], *ε*_InAs_ = 12.8 [37]), while the wavelength of the pump wave is 2.07 μm (*ε*_Au_ = −225 + 7.8*i* [24], *ε*_InAs_ = 11.9 [37]). (**c**) Simulated current-voltage characteristics of the device shown in panel (**a**) at different optical pump power densities. (**d**,**e**) Energy band diagrams of the device at bias voltages of 0.2 V (panel (**d**)) and 0.4 V (panel (**e**)) in the vicinity of the Au/InAs interface in the absence and presence (*P*_pump_ = 0.5 mW/μm^2^) of optical pumping. *E*_c_ and *E*_v_ denote the band edges of the conduction and valence bands, respectively. (**f**,**g**) Spatial distribution of the electron (panel (**f**)) and hole (panel (**g**)) densities in the vicinity of the Au/InAs interface at three bias voltages in the absence and presence (*P*_pump_ = 0.5 mW/μm^2^) of optical pumping. Abbreviations: SPP, surface plasmon polariton.

Figure 1a shows a schematic illustration of the metal-semiconductor Au/InAs planar plasmonic waveguide. The thickness of the InAs layer is chosen to be twice larger than the penetration depth of the electromagnetic energy of the SPP guided by the Au/InAs interface (Figure 1b) to minimize the impact of the thickness of the InAs layer on the SPP characteristics, which allows us to analyze the hybrid pumping approach without diminishing the generality of the obtained results. The Au/InAs contact gives the possibility to inject electrons directly from gold to the *p*-type-doped InAs layer (the concentration of acceptors is *N*_A_ = 2.3 × 10^18^ cm^−3^) and pump the structure electrically [26]. To avoid the complexity of the structure and investigate the fundamental properties of the hybrid pumping, we assume the top contact in Figure 1b to be an ideal ohmic contact, which might be realized by using a transparent electrode [36]. However, we note that for practical applications, the usage of a heterojunction [24,32] is more reasonable. The structure can also be pumped optically. The operating free-space light wavelength is chosen to be equal to *λ*_0_ = 3.26 μm (*ћω*_0_ = 0.38 eV), which roughly corresponds to the wavelength at which the maximum optical gain in the InAs can be achieved in the regime of full loss compensation, and is dictated by the bandgap energy of InAs [26]. If there is no loss or gain in the InAs layer, the SPP propagation length is equal to *L*_SPP_ = 52 μm (*ε*_Au_ = −561 + 30*i* [24], *ε*_InAs_ = 12.8 [37] at *λ*_0_ = 3.26 μm). InAs can also be pumped optically at a free-space light wavelength of about 2 μm (*ћω*_p_ = 0.6 eV). Such a short pump wavelength allows the generation of electron-hole pairs even at a high degree of population inversion in InAs. The high density of electron-hole pairs created in InAs by electrical pumping and optical pumping gives the possibility to compensate for the SPP propagation losses and even amplify the SPP.

To simulate the SPP amplification under hybrid electro-optical pumping, we employed a self-consistent model that comprises the Poisson equation for the static electric field and carrier densities, the drift-diffusion equations for free electrons and holes, and the carrier continuity equations, which include non-radiative Auger recombination, spontaneous emission into the plasmonic mode and into free space, stimulated emission of SPPs, and absorption of the pump radiation:

(1) $$\{\begin{array}{ccc} \frac{d\varphi }{dz} & = & -\varepsilon ,\\ \frac{d\varepsilon }{dz} & = & \frac{4\pi e(p-n-N_{A})}{\varepsilon_{\mathrm{st}}},\\ j_{n} & = & eD_{n}\frac{dn}{dz}+e\mu_{n}n\varepsilon ,\\ j_{p} & = & -eD_{p}\frac{dp}{dz}+e\mu_{p}p\varepsilon ,\\ \frac{dj_{n}}{dz} & = & e(R_{\mathrm{spont}}+R_{\mathrm{stim}}+R_{\mathrm{nr}}+R_{\mathrm{opt}}),\\ \frac{dj_{p}}{dz} & = & -e(R_{\mathrm{spont}}+R_{\mathrm{stim}}+R_{\mathrm{nr}}+R_{\mathrm{opt}}). \end{array}$$

In these equations, which are solved using Newton’s method, *φ* is the electrostatic potential, *ε* is the static electric field, *e* is the elementary charge, *n* and *p* are the densities of electrons and holes, respectively, *j*_n_ and *j*_p_ are the electron and hole electric current densities, *D*_n_ and *D*_p_ are the diffusion coefficients, *μ*_n_ and *μ*_p_ are the electron and hole mobilities. Finally, *R*_spont_, *R*_stim_, *R*_nr_, *R*_opt_ are the rates of spontaneous emission, stimulated emission, nonradiative Auger recombination, and recombination associated with optical pumping, respectively.

We determine *R*_spont_ via the Einstein relation between the spontaneous and stimulated emission processes:

(2) $$R_{\mathrm{spont}}(z)=\int P(\omega ,z)g\left(\omega ,n(z),p(z)\right)n_{\mathrm{sp}}(\omega ,z)\rho_{3D}(\omega )d\omega ,$$

where *g* is the material gain, *P* is the Purcell factor, $\rho_{3D}(\omega )=\omega^{2}n_{\mathrm{InAs}}^{3}(\omega )/(\pi^{2}c^{3})$ is the photonic density of states in a bulk material. The spontaneous emission factor *n*_sp_, given by

(3) $$n_{\mathrm{sp}}(\omega ,z)=\frac{1}{1-\exp\left(\frac{ћ\omega -(F_{e}(z)-F_{h}(z))}{k_{B}T}\right)},$$

characterizes the degree of population inversion. In Equation (3), *F*_e_ and *F*_h_ are the quasi-Fermi levels for electrons and holes, respectively, and *k*_B_*T* is the thermal energy. The spontaneous emission rate *R*_spont_ accounts for the enhanced spontaneous emission due to the existence of the guided plasmonic mode strongly-confined to the metal-semiconductor interface. The Purcell factor is evaluated as [38]

(4) $$P(\omega ,z)=P_{\mathrm{free}}(\omega ,z)+\frac{\beta (\omega )n_{g}(\omega )}{16{\left(\frac{\omega }{c}\right)}^{2}n_{\mathrm{InAs}}(\omega )}\frac{|E(\omega ,z){|}^{2}}{\int_{-\infty }^{\infty }\left[w_{E}(\omega ,z)+w_{M}(\omega ,z)\right]dz},$$

where *P*_free_(*ω*,*z*) is the Purcell factor associated with emission into free space modes, *β* is the wavenumber of the SPP, *n*_g_ is its group index, *n*_InAs_ is the refractive index of InAs, *w*_E_ and *w*_M_ are the electric and magnetic energy densities of the SPP, ***E***(*z*) is the complex amplitude of the SPP electric field. In our simulations, we assumed that *P*_free_(*z*) ≈ 1. In the studied waveguide geometry, the Purcell factor is as large as 2 near the Au/InAs interface and decreases as the distance from the interface increases. The stimulated emission recombination rate *R*_stim_ is a function of the SPP power density per unit waveguide width *P*_SPP_ and the spatial distribution of the material gain in InAs:

(5) $$R_{\mathrm{stim}}(z)=\frac{P_{\mathrm{SPP}}}{ћ\omega_{0}}\frac{n_{g}(\omega_{0})}{n_{\mathrm{InAs}}(\omega_{0})}g(\omega_{0},n(z),p(z))\frac{2w_{E}(\omega_{0},z)}{\int_{-\infty }^{\infty }[w_{E}(\omega_{0},z)+w_{M}(\omega_{0},z)]dz}$$

Finally, the rate of recombination associated with optical pumping is given by

(6) $$R_{\mathrm{opt}}(z)=-\frac{c\alpha (\omega_{p},n(z),p(z))}{n_{\mathrm{InAs}}(\omega_{p})ћ\omega_{p}}2w_{E}^{\mathrm{pump}}(z),$$

where *n*_InAs_(*ω*_p_) = 3.45 is the refractive index of InAs at the pump wavelength, *α*(*ω*_p_, *n*(*z*), *p*(*z*)) = −*g*(*ω*_p_, *n*(*z*), *p*(*z*)) is the material loss of InAs at the pump wavelength, and $w_{E}^{\mathrm{pump}}$ is the electric energy density of the pump wave, which is very non-uniform in the InAs layer due to the reflection from the InAs/Au interface and formation of a standing wave (Figure 1b). The material gain used in Equations (2), (5), and (6) is a function of the photon energy and the electron and hole densities and is calculated in every point of the InAs layer at every simulation step using the approach presented in [39].

We emphasize that the developed theoretical approach simultaneously takes into account the non-zero power of the SPP wave, the non-uniform distribution of the SPP electromagnetic field in the waveguide cross-section, and the spatial distribution of the electromagnetic field of the pump optical wave, which cannot be done with the commercial simulation software packages.

The results of the numerical simulations of the hybridly pumped planar plasmonic waveguide at *T* = 77 K are presented in Figure 1c–e. The temperature is set to *T* = 77 K due to the strong nonradiative recombination in InAs at temperatures above 100–150 K [40,41]. The current-voltage characteristics for different optical pump power densities *P*_pump_ show the same typical diode-like behavior with a turn-on voltage of about 0.35 V (Figure 1c). However, it should be noted that as *P*_pump_ increases, the absolute value of the current density at *V* < 0.35 V rapidly increases and can be as high as 30 kA/cm^2^. The optical pumping generates free carriers in InAs, and the electrons flow towards the bottom gold contact (negative electrode), and the holes flow towards the top ohmic contact (positive electrode), which results in a negative current. Such unusual directions of the carrier flow are determined by the band bending of InAs at voltages below about 0.4 eV (Figure 1d). Since the carrier recombination rate is much lower than the carrier generation rate, the current almost does not depend on the bias voltage at *V* < 0.35 V. However, as the bias voltage increases, the current increases and eventually becomes positive (Figure 1c) at voltages above ~0.4 V, which is due to the decrease of the strength of the “photodetection” effect due to flattening of InAs bands (Figure 1e) and increase in the electron and hole forward currents with the increase in the bias voltage.

The Fermi level in gold lies 0.13 eV above the conduction band edge of InAs [42], which provides a very thin “inversion” layer near the Au/InAs contact where the electron density is as high as 10^18^ cm^−3^ (Figure 1f), while in the bulk of InAs, the electron density is more than ten orders of magnitude lower in equilibrium. In the absence of optical pumping, under high forward bias (*V* ≳ 0.35 eV), electrons are efficiently injected into the bulk of p-type InAs (Figure 1f), which allows to create population inversion in InAs required for the amplification of SPPs [26,32]. If the structure is also pumped optically, the spatial distribution of the electron density is significantly different. The inversion layer near the Au/InAs interface also exists; however, the electron density in the bulk of InAs is determined by the optical pumping and is as high as 2 × 10^15^ cm^−3^ at *P*_pump_ = 0.5 mW/μm^2^ and bias voltages below *V* ≈ 0.35 V. Only at higher bias voltages, the strong electron injection from gold gives the possibility to increase the electron density. The hole density, which is much higher than the electron density in the *p*-type-doped InAs layer, is not sensitive to optical pumping (Figure 1g), which explains the same diode-like behavior for the current-voltage characteristics under different optical pumping levels. The spatial distribution of holes is determined only by the change in the thickness of the depletion region near the Au/InAs interface, which decreases as the bias voltage increases (Figure 1g).

**Figure 2:**
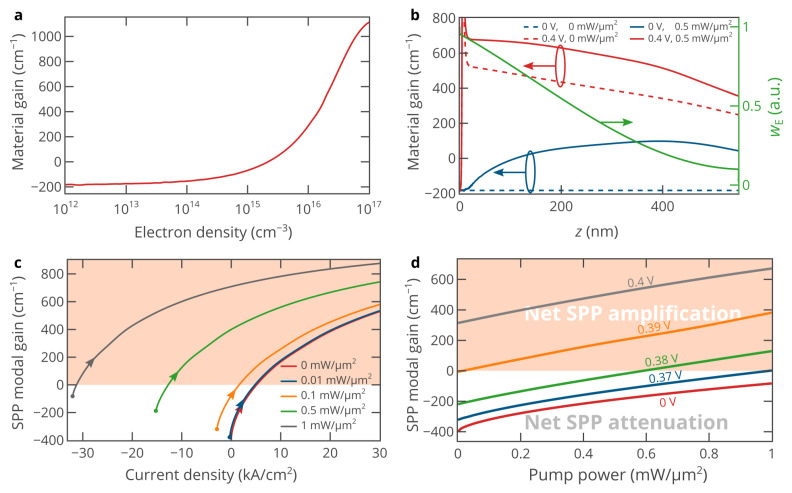
(**a**) Material gain of *p*-type-doped InAs at a free-space light wavelength of 3.26 μm as a function of free electron density calculated employing Stern’s model for interband transitions in heavily-doped semiconductors [39,44], which accounts for the band tails in the density of states arising due to a chaotic screened potential of charged dopants. (**b**) Spatial distribution of the material gain across the InAs layer at bias voltages of 0 and 0.4 V in the presence and absence of optical pumping (0.5 mW/μm^2^). To estimate the overlap of the gain and mode profiles, the spatial distribution of the electric energy density of the SPP field is also shown. (**c**) SPP modal gain as a function of the current density at different levels of optical pumping. The dots on the curves correspond to *V* = 0 V, while the arrows show the direction of the bias voltage increase. (**d**) SPP modal gain as a function of the optical pump power density at five different bias voltages. In panels (**c**) and (**d**), the region that corresponds to the net SPP amplification is shown in red.

The material gain in InAs *g*(*ω*_0_, *n*, *p*) is mostly determined by the electron density distribution since the hole density is almost the same across the InAs layer at any bias voltage and optical pump power density. At a very low electron density, *g*(*ω*_0_) is about −180 cm^−1^ (Figure 2a), and it increases as *n* increases. At *n* = 2.2 × 10^15^ cm^−3^, InAs becomes transparent, and accordingly, if one creates a higher electron density, it is possible to compensate for the SPP propagation losses. Figure 2b shows the spatial distribution of the material gain at two bias voltages in the presence and absence of optical pumping. At *V* < 0.3 V and *P*_pump_ = 0, the material gain is equal to −180 cm^−1^ over the whole InAs layer due to the absence of pumping. The optical pumping at a power density of *P*_pump_ = 0.5 mW/μm^2^ creates gain in InAs (Figure 2b) and make it almost transparent for the SPP propagating along the Au/InAs interface, since the modal gain *G* of the SPP mode is given by [43]:

(7) $$G=G_{\mathrm{InAs}}-\alpha_{\mathrm{SPP}}=\frac{n_{g}}{n_{\mathrm{InAs}}(\omega_{0})}\frac{\int 2w_{E}(z)g(\omega_{0},z)dz}{\int \left[w_{E}(z)+w_{M}(z)\right]dz}-\alpha_{\mathrm{SPP}},$$

where *α*_SPP_ = 1/*L*_SPP_ = 190 cm^−1^ is the SPP modal loss at an optically transparent InAs. Equation (7) shows that the SPP modal gain is determined by the spatial distribution of material gain in InAs and its overlap with the spatial distribution of the electric field of the SPP mode. At a very high forward bias voltage, the material gain of InAs is primarily determined by electrical pumping (Figure 2b), and the contribution of optical pumping to the net SPP modal gain is lower than at *V* = 0 due to the nonlinear dependence of the material gain and non-radiative recombination on the electron density, which reduces population inversion faster at a higher free carrier density. Thus, the hybrid electro-optical pumping cannot be considered as a linear superposition of the electrical pumping and optical pumping.

Figure 2c shows the dependence of the SPP modal gain on the injection current for five different levels of optical pumping. For the selected optical pump powers, at *V* = 0, the net SPP gain is negative, i.e., the SPP loses energy when propagating. However, as the current (bias voltage) increases, the modal gain increases and eventually becomes positive (net SPP amplification). As the optical pump power increases, the current-gain curve shifts to the left since a lower injection current is required to fully compensate for the SPP propagation losses at a higher optical pump power (Figure 2c). At zero voltage (the short-circuit condition), the full SPP loss compensation is achieved at an optical pump power density of as high as 1.5 mW/μm^2^. Under such strong optical pumping, the current density at zero voltage (the short-circuit condition) is as high as −50 kA/cm^2^, which significantly reduces population inversion in InAs. Therefore, it is more efficient to operate under the open-circuit condition (at *j* = 0 and non-zero bias voltage) (Figure 2c), i.e., under pure optical pumping, since a much higher SPP modal gain can be achieved. However, a significantly higher gain can be achieved at *j* > 0, which is one of the advantages of hybrid pumping. Under hybrid electro-optical pumping, the SPP modal gain at high forward currents (high forward bias voltages) can be several times higher than at *j* = 0. Thus, electrical pumping can enhance optical pumping. Similarly, optical pumping can be used to control and enhance electrical pumping, as shown in Figure 2d. From the practical point of view, it is easier to control the bias voltage rather than the injection current. Since the turn-on voltage of the considered diode is about 0.35 V, the voltage of at least 0.36 V should be applied (Figure 2d).

One of the most attractive features of hybrid electro-optical pumping is the possibility to operate at current densities about zero. At *j* = 0, the gain is provided only by optical pumping. However, by slightly decreasing the bias voltage, one makes the current negative, and therefore, reduces the SPP modal gain. Accordingly, by slightly increasing the bias voltage, one increases the SPP modal gain. Since the optical pumping approach is not scalable and compact, this feature allows to precisely tune the modal gain at any given point of a large-scale plasmonic circuit or tune a plasmonic laser. It is remarkable that in this case, the power consumption *jV* of the external electronic circuit that controls electrical pumping is very low due to the low electric current *j*. Similarly, it is possible to precisely tune the modal gain around a higher value that corresponds to a higher current density. It is interesting that at negative current densities, the DC power source of the electronic circuit that controls electrical pumping does negative work. In other words, this process can be used to charge the battery that powers the electrical pumping circuit.

**Figure 3:**
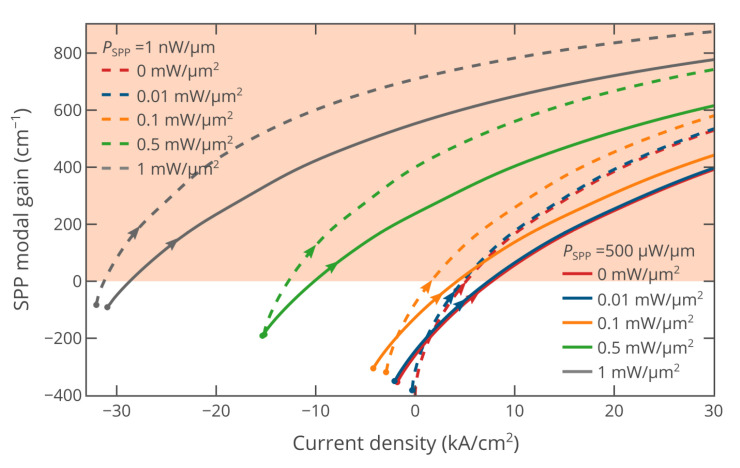
SPP modal gain in the hybridly pumped active plasmonic waveguide as a function of the current density at different levels of optical pumping for the SPP powers of 500 μW/μm (solid lines) and 1 nW/μm (dashed lines). The dots on the curves correspond to *V* = 0 V, while the arrows show the direction of the bias voltage increase.

The above simulations were performed at a very low SPP power per unit waveguide width of 1 nW/μm. Such a low SPP power almost does not affect the densities of nonequilibrium electrons and holes in InAs, i.e., the stimulated emission recombination rate *R*_stim_ (see Equations (1) and (5)) is negligibly small. However, in practical applications, the SPP power cannot be equal to zero. Moreover, the SPP power can be quite high. For example, in on-chip communication, each optical pulse must carry a non-zero amount of energy to be efficiently detected at a photodetector and not to be significantly distorted during propagation. Assuming this energy to be of about 1–5 *fJ* [45,46], the bit rate to be of the order of 100 Gbit/s, and the waveguide width to be of about 300 nm [24,39], we obtain an average SPP power per unit waveguide width of as high as 150–750 μW/μm. Thus, in a practical system, the SPP power density is comparable with the power density of optical pumping, which can strongly affect the possibility of compensating for the SPP propagation losses. Figure 3 shows the SPP modal gain at an SPP power per unit waveguide width of 500 μW/μm. It can be seen that at zero optical pump power and zero bias voltage, the current is negative, which is due to the “photodetection” effect. At *V* = 0 and *P*_pump_ = 0, the material gain in InAs is negative, and therefore, it absorbs the SPP electromagnetic field. Hence, the curves at a high SPP power are left shifted compared with curves at nearly zero SPP power. The slopes of the curves are lower than at *P*_SPP_ = 1 nW/μm, since it is more difficult to create gain in InAs at a higher SPP power due to the stronger depopulation of electron and hole densities in InAs through the stimulated emission (see Equations (1) and (5)). However, the high SPP power does not prevent us from achieving full compensation for the SPP propagation losses.

**Figure 4:**
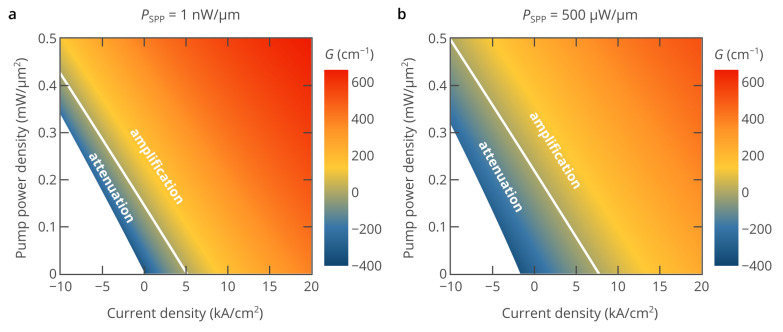
Heatmap of the SPP modal gain as a function of the pump current and optical pump power for a low-power signal (*P*_SPP_ = 1 nW/μm) (panel (**a**)) and a high-power signal (*P*_SPP_ = 500 μW/μm) (panel (**b**)). In both panels, the white line corresponds to the regime of lossless SPP propagation, i.e., the net SPP modal gain is equal to zero, and the propagation losses are fully compensated by gain in InAs. The white area in the left bottom corner corresponds to negative bias voltages, which are out of practical interest for hybrid pumping.

While Figure 2 and Figure 3 show the dependence of the SPP modal gain only on either the pump current or optical pump power, the hybrid pumping approach can be better understood by considering the dependence of the SPP modal gain on both electric pump current and optical pump power (Figure 4). Both the pump current and optical pump power can be continuously varied, which allows to freely select the desired regime of SPP amplification. It can be seen that the same level of SPP amplification can be achieved at different combinations of optical and electrical pumping. In particular, the regime of lossless SPP propagation, when the SPP is neither amplifier nor attenuated, is indicated by the white line. It is interesting that although the gain-current curves in Figure 2 and Figure 3 show clear signs of saturation at high pump currents, the lines of the same SPP modal gain at a fixed SPP power in Figure 4 are almost parallel to each other. This means that the reduction in the efficiency of both electrical and optical pumping with the increase in the pump current or pump power is mostly due to the nonlinear dependence of the material gain of InAs and the Auger recombination rate on the free carrier densities.

## 3. Conclusions

We present a comprehensive study of surface plasmon polariton amplification under hybrid electro-optical pumping in Au/InAs metal-semiconductor structures. Using a self-consistent numerical approach, we demonstrate that under simultaneous electrical and optical pumping, it is possible to achieve much higher SPP modal gain than under pure electrical or pure optical pumping. At the same time, we find that the gain provided by hybrid pumping cannot be represented as a linear superposition of optical and electrical pumping due to the strongly nonlinear dependence of the material gain of the semiconductor and the Auger recombination rate on the density of nonequilibrium free carriers. Using the hybrid pumping approach, it is possible to achieve a better performance than using only optical or only electrical pumping. For example, in spite of its convenience in laboratory conditions, optical pumping is not scalable and is difficult to be used in high-density optoelectronic circuits since it is almost impossible to tune the modal gain locally. The hybrid pumping approach can easily solve this problem. By slightly increasing or decreasing the pump current, one can precisely adjust the SPP modal gain at any given point of the large-scale plasmonic circuit. It is remarkable that such adjustments can be made by slightly varying the pump current density around zero. Thus, even if the structure cannot provide high gain under pure electrical pumping, it is still possible to tune (increase or decrease) the gain created by optical pumping. At the same time, the power consumption *jV* of the external electronic circuit that controls electrical pumping is extremely low, which makes such a tuning approach promising for many practical applications. One of many advantages of local tuning of the SPP modal gain is the possibility to reduce the spontaneous emission noise, which is unavoidable in structures with gain [38]. By redistributing the SPP modal gain along the active plasmonic waveguide and making it non-uniform, it is possible to improve the signal-to-noise ratio by more than 100% [47]. Similarly, the hybrid approach can be used for dynamic tuning of plasmonic nanolasers. Moreover, if the nanolaser is compatible with hybrid electro-optical pumping, it can operate under pure electrical pumping, while pure optical pumping can be used to charge the battery that powers the nanolaser, which is an attractive feature for nanoscale optoelectronic devices for different biological applications [48]. To conclude, our findings provide insights into the development of electro-optically pumped active plasmonic devices and demonstrate the advantages of this hybrid approach.
